# Recommendations to facilitate self-care practices among professional nurses

**DOI:** 10.4102/curationis.v48i1.2755

**Published:** 2025-12-16

**Authors:** George J. Nkabinde-Thamae, Charlene Downing

**Affiliations:** 1 Department of Nursing, Faculty of Health Sciences, University of Johannesburg, Johannesburg, South Africa

**Keywords:** self-care, practices, recommendations, professional nurses, primary healthcare, physical activity, lifestyle, strategies

## Abstract

**Background:**

Nursing is regarded as a caring profession, with a core focus on providing care to others to improve or maintain their health. As a result, ongoing research is conducted to provide recommendations for improved clinical outcomes, which professional nurses would usually execute. However, despite the continuous changes and challenges within the nursing profession, little effort has been made to unveil explicit guidance on how professional nurses should engage in self-care practices to promote and sustain their well-being.

**Objectives:**

This study aimed to describe recommendations to facilitate professional nurses’ empowerment in practising self-care as a lifestyle.

**Method:**

A qualitative, explorative, descriptive and contextual research design was employed to obtain rich data from 10 purposively sampled professional nurses. Unstructured individual interviews were conducted and transcribed verbatim. Data were analysed using Colaizzi’s method.

**Results:**

The main study uncovered three themes, but this study explored only one: professional nurses’ urgent need to break free from the vicious, depriving cycle of not practising self-care.

**Conclusion:**

Sustainable recommendations are needed to empower professional nurses in self-care practices.

**Contribution:**

This study provides clear and cost-effective recommendations on what professional nurses can do to promote self-care practices.

## Introduction

Mills, Wand and Fraser (2018) assert that self-care practices among professional nurses are regarded as an active, holistic process performed by individuals to promote their health and well-being. Thus, this study underpinned Orem’s self-care framework as it unveiled the importance of self-care and relevant actions of an individual to promote their health and overall well-being (Orem 2016). Despite the importance of self-care practices, many professional nurses seem to struggle with them; therefore, interventions are necessary to support these nurses in practising self-care (Millenbach et al. 2023). In addition, various researchers have unveiled that the self-care phenomenon has been explored in different healthcare settings. Wei et al. (2020) focused on self-care and burnout among paediatric critical care nurses and physicians. Moreover, Wei et al. (2019) contributed to the phenomenon by exploring strategies to foster resilience among professional nurses and nurse managers in a general hospital setting. Lastly, Nkabinde-Thamae, Downing and Nene (2022), Muhlare and Downing (2023) and Nkabinde-Thamae and Downing (2024) provided a vivid snapshot of the experiences of professional nurses employed in primary healthcare environments regarding self-care practices. These independent studies indicate the emphasis on self-care practices among professional nurses in various clinical settings and reveal that professional nurses face challenges in engaging in self-care practices.

Understanding the extent of self-care neglect in the world of professional nurses, the researchers realised the importance of recommendations aimed at enhancing self-care practices among nurses to make self-care practices achievable. LoBiondo-Wood and Haber (2018) recommend that qualitative studies apply surveys to technique, theory and future research. Thus, the researchers in this study believe that specific, transparent and sustainable recommendations are vital for guiding professional nurses in incorporating self-care practices into their daily routines to enhance their well-being. Nicholls et al. (2016) advise that healthcare institutions should develop and implement sustainable strategies or recommendations to assist professional nurses in overcoming possible barriers to self-care practices. Furthermore, Ross et al. (2019) state that strategies to help nurses with self-care practices should include interventions that deal with how to overcome the lack of time to practise self-care, being able to say no to situations that may drain the individual nurse and attempting to engage in healthy eating habits. Cook-Cottone and Guyker (2018) assert that physical care, good sleeping patterns, regular exercise routines and nutritious meals are fundamental to supporting individual nurses with self-care practices. The tentative perceived strategies for promoting self-care practices in the world of professional nurses are more comprehensive than the above-mentioned content. In relation to this study, the researchers will provide clear and specific recommendations based on the insights shared by participants during the data collection process regarding what they considered feasible for practising self-care, after recognising the extent to which they had neglected self-care and its impact.

### Problem statement

Millenbach et al. (2023) state that professional nurses employed in healthcare settings struggle to create and maintain a balance between the demands of the profession and the practice of self-care, which negatively affects their well-being. In addition, LoGiudice and Bartos (2021) affirm that professional nurses struggle with self-care practices; their focus is on providing care to their patients. Muhlare and Downing (2023) noted that professional nurses in primary healthcare facilities struggle with self-care practices, primarily because of a lack of consciousness of their health, inadequate sleep of fewer than 8 h, unhealthy dietary practices and physical inactivity. One of the researchers is employed in a primary healthcare environment and has observed similar practices among professional nurses. In a social conversation with them, the professional nurses shared that they prioritised others, such as patients and family, but did not prioritise self-care practices. A study done by Wong et al. (2018) concurs that professional nurses do not prioritise their health needs as their daily desire is to provide care to their patients and individual family members. The literature concludes that healthcare organisations must assist professional nurses in incorporating self-care practices into their daily routine (Bermejo-Martins et al. 2021).

### Purpose

This study aimed to explore and describe recommendations to facilitate professional nurses’ empowerment to practise self-care as a lifestyle.

## Research methods and design

A qualitative descriptive approach was utilised to address the aim of the study (Doyle et al. 2019). In addition, the researchers felt that an exploratory qualitative design would be appropriate to enable the professionals to share their experiences regarding the phenomenon (Polit & Beck 2020). Hennink, Hutter and Bailey (2020) and Polit and Beck (2021) affirm that qualitative, descriptive and contextual research discloses the importance of understanding the whole, which requires the researcher to be involved and employ an interpretative approach.

### The setting of the study

The study was conducted in 10 primary healthcare facilities within the eastern sub-district of the City of Ekurhuleni, located in Gauteng province. The total number of healthcare facilities is 25, operating from Monday to Friday, between 8:00 to 16:30 (Nene & Wopula 2025; Wopula, Nene & Nkosi 2022). Bhandari et al. (2020) state that the type of healthcare services provided at these healthcare facilities are for outpatients in need of treatment and care for common illnesses and are provided at no cost. Primary healthcare provides preventive services, such as antenatal care (ANC), postnatal care (PNC), immunisations, family planning and acute and chronic health services (Kapologwe et al. 2020). The rationale for selecting this research setting is stated in the problem statement of this study. Furthermore, Bruno, Aquino and Pringsheim (2024) advise that a research setting should be selected based on the research questions, objectives, ethical considerations and the desired time to complete the research study.

### Population and sampling strategy

The research population consisted of professional nurses employed within the Ekurhuleni Eastern sub-district. According to Casteel and Bridier (2021) and Majid (2018), a population should include all appropriate individuals or groups of people who are of interest to the researcher or who can contribute to the phenomenon. Creswell and Poth (2018) and Hennink et al. (2020) assert that purposive sampling refers to selecting participants who possess knowledge about the research topic to ensure the richness of data. Therefore, a purposive sampling method was deemed appropriate for this study to allow participants to participate based on their knowledge and lived experience of the phenomenon. This was a qualitative study; therefore, the sample size was determined by data reaching saturation at the 10th interview. This is supported by the findings by Hennink and Kaiser (2022), which stated that data are generally reached by the 9th or 17th interview, where no new themes or sub-themes emerge.

### Inclusion and exclusion criteria

The inclusion criteria for this study required participants to be professional nurses employed for at least a year within a primary healthcare facility. The rationale for stipulating minimum work experience was to ensure that participants were able to provide relevant accounts of day-to-day work experience within primary healthcare facilities. In addition, participants had to be over 18 years of age and reside within Gauteng province to provide written consent. However, participants residing outside Gauteng province were excluded from the study.

### Data collection

Data collection was conducted through individual, unstructured interviews. Creswell (2014) states that unstructured interviews aim to understand the phenomenon through the participants’ views and knowledge. In this study, the primary author conducted all the interviews within 4 months, between December 2020 and March 2021. All interviews were conducted in English and lasted between 45 min and 60 min. A total of 10 professional nurses from 10 different primary healthcare facilities consented to participate in this study; the 10 participants comprised of 5 females and 5 males, each with work experience ranging from 4 to 25 years in a primary healthcare facility (Nkabinde-Thamae & Downing 2024). Apart from the 10 professional nurses who participated, a total of 7 professional nurses were unable to take part in the study. Two professional nurses did not meet the inclusion criteria, two wished to participate after the data analysis was completed, and three professional nurses showed interest after the main study had concluded.

Furthermore, data collection continued until data saturation was reached, at which point no new themes or sub-themes emerged from the data, as recommended by Gray and Grove (2020). To ensure the recruitment of relevant participants for this study, the primary researcher arranged a meeting with the Senior Manager of the region, the Nursing Manager and Chief Professional Nurses after obtaining all relevant ethical and gateway approvals. The purpose of the meeting was to explain the study to them and to allow them to ask any questions for clarification, but during the meeting, no concerns were raised. After the scheduled meeting, the primary researcher distributed flyers and posters at all the approved healthcare facilities for participants who were interested in participating in the study and who met the inclusion criteria. All 10 interviews were arranged with all participants at a location and time that was convenient for them, ensuring that service delivery was not disrupted. The main study had two objectives: to explore and describe professional nurses’ lived experiences of practising self-care and to provide recommendations to facilitate professional nurses’ empowerment in practising self-care as a lifestyle (Nkabinde-Thamae 2021). Each objective was explored by asking one central question: *What would the participants do to practise self-care?* Further classification was explored through effectively applying communication skills, such as the classification of open-ended questions. The interview guide as detailed in Table 1 gives further insight into the probing questions asked.

**TABLE 1:** Interview guide.

At the beginning of the interview, you stated that there are several factors preventing you from engaging in self-care practices. Let’s talk more about that.
Daily activities such as walking or reading a book sound like basic activities; how will these activities assist you in practising self-care?
You mentioned sleep; how will an adequate sleep routine contribute to self-care practices?
You seemed sad when you mentioned diet. What prevents you from having a nutritious diet daily?
Which of these mentioned interventions are a priority to you?
Let’s talk more about how self-care neglect has an impact on your mental well-being.
Working overtime seems to be depriving you from engaging in self-care practices; let’s talk more about that.

### Data analysis

Data were analysed using Colaizzi’s seven-step method (Colaizzi 1978; Converse 2012; Praveena & Sasikumar 2021), rooted in Husserl’s phenomenology approach. In Step 1, the primary researcher transcribed all 10 recordings verbatim, and the transcriptions were read repeatedly to ensure that the information captured during data collection was a true reflection. In Step 2, the participants described the reality of not practising self-care and explored with the primary researcher what could be done to encourage self-care practices in their personal and professional lives. This allowed the researchers to gain an understanding and meaning of the phenomenon from the perspective of the participants. In Step 3, the primary researcher interpreted the obtained content and clarified any ambiguities with the relevant participants to formulate meaning. In Steps 4 and 5, 1 theme and 5 sub-themes were assigned. In Step 6, both researchers returned to all verbatim transcripts to confirm whether the themes and sub-themes presented the proposed recommendations on self-care practices. In the last step, Step 7, the researchers communicated to all the participants all the identified themes and sub-themes to validate the findings. This was done through member checking to determine whether the findings presented the desired strategies regarding recommendations for self-care practices.

### Ethical considerations

Ethical clearance was obtained from the University of Johannesburg Faculty of Health Sciences and the Research Ethics Committee (REC-241112-035). Further permission to conduct the study was required from the City of Ekurhuleni Research Ethics Committee (11/06/2020-01) and the National Health Research Database (GP_202005_032). As advised by Polit and Beck (2021), the researchers upheld ethical principles throughout the study. The principle of autonomy was upheld by explaining the study’s aims, objectives and the entire research process to all participants, which empowered them to make an informed decision whether they wanted to participate or not. The primary researcher emphasised that they were allowed to withdraw from the study at any time. Furthermore, the principles of beneficence and non-maleficence were promoted by ensuring that no harm or discomfort was deliberately caused to participants; thus, the primary researcher displayed sensitivity when asking clarifying or probing questions. Participation was entirely voluntary; thus, the researchers did not coerce any of the participants. Those who chose to participate met the inclusion criteria after attending the information session and providing written consent to participate in the study after they had a complete understanding of what the study entails. Lastly, confidentiality was upheld by the primary researcher conducting all interviews in locations convenient for the participants yet separate from their workplace, allowing them to freely express themselves, without any fear of reprisal. Anonymity was also upheld by ensuring that participants’ identities were not disclosed during or after the study. All data collected are kept in a secure location under lock and key, and password protected.

### Trustworthiness

To ensure trustworthiness, the researchers employed the five principles of Lincoln and Guba: credibility, transferability, dependability, conformability and authenticity (Grove & Gray 2019; Korstjens & Moser 2018). The researchers promoted credibility through extended engagement with the participants. The primary researcher spent sufficient time with the participants, between 45 min and 60 min, to develop a rapport, mutual trust and respect. Member checking was conducted after the data analysis and was shared with all 10 participants to determine the accuracy of the findings. Dependability was accomplished by ensuring that the data were coded over an extended period to ensure consistency in coding. In addition, an independent coder with experience in qualitative studies was employed; the independent coder and the primary researcher analysed the data independently and met to reach a consensus. Transferability was achieved through a clear description of the research methodology. Confirmability was achieved by transcribing the recorded information verbatim and documenting direct quotations from the 10 participants.

## Results

This study aimed to explore and describe recommendations to facilitate professional nurses’ empowerment to practise self-care as a lifestyle. The results revealed a clear narrative on what needs to be done to make self-care practices a reality in the lives of professional nurses. From the main study, three themes emerged: internal and external factors compromising self-care practices, the impact of self-care neglect on patients’ and nurses’ holistic well-being, and lastly, the participants expressed a need to take responsibility and be accountable for not practising self-care. However, for the purposes of this study, only the results related to theme 3 will be discussed, as this theme supports the intended contribution to this study (Table 2). During the data collection process, it became evident to the researchers that the participants struggled with self-care practices, which resulted from a lack of time to engage in basic activities, such as reading a book or listening to their favourite music. Engaging in unhealthy eating habits and not having time to engage in physical activities, such as going to the gym or exercising in general, has led some to complain of undesirable weight gain. In addition, the participants worked overtime to supplement their income, depriving themselves of adequate sleep and leaving them feeling fatigued and unable to cope with their day-to-day duties. The recommendations will stem from the content as the participants shared their intentions to promote self-care practices in their daily lives. Thus, recommendations for this study entailed strategies shared by the participants.

**TABLE 2:** Theme and sub-themes.

Theme	Sub-themes
The participants experienced an urgent need to break free from this vicious, depriving cycle of not practising self-care and realised that they had to take up the responsibility themselves to practise self-care.	Engage in personal activities (reading books, listening to favourite music) to promote self-care practices. The promotion of adequate sleeping patterns by cutting on overtime. Practise healthy eating habits to promote self-care practices. Promoting self-care practices by engaging in physical activity. To promote mental wellness through debriefing and employee wellbeing programmes.

*Source*: Nkabinde-Thamae, G.J., 2021, ‘Practices of self-care by professional nurses working at a primary health care clinic in Gauteng’, Unpublished master’s dissertation, University of Johannesburg

### Theme: The participants experienced an urgent need to break free from this vicious, depriving cycle of not practising self-care and realised they had to take up the responsibility to practise self-care

This theme revealed the participants’ realisation of what they needed to do to practise self-care. Their recommendations would become strategies for clear, cost-effective interventions to assist them in developing self-care practices. Despite the various contributors to self-care neglect, the participants were eager to start somewhere and take accountability and responsibility.

#### Sub-theme 1: Engage in personal activities (reading books, listening to favourite music, relaxing techniques) to promote self-care practices

The participants shared interventions that seemed to be quick wins and within their means, as they appeared to be interventions that did not need money. In addition, the following verbatim quotes indicate that even if these interventions seem basic and simple, the participants were not engaging in these activities on a regular basis:

‘I have been neglecting myself and taking care of everyone, so going forward, I will try to practise self-care. There is a lot I would want to do, but I will start with activities that I think will be a bit easy, like listening to music to relax my mind a little.’ (Participant 3, 45 years old, female)

Two other participants shared:

‘To be honest, self-care practices do not need money, so from today on, I will just shut down my mind when I take a bubble bath to relax and stop overthinking and worrying about work-related stuff, like staff shortage.’ (Participant 2, 34 years old, male)

‘I normally feel overwhelmed because of the work-related stress, and maybe because of my own personal stressors, but meditating and starting a small garden at home will ask me to clear my mind.’ (Participant 5, 51 years old, female)

#### Sub-theme 2: The promotion of adequate sleeping patterns by cutting on overtime

The preceding findings provide insight into what contributes to professional nurses not engaging in self-care practices, and it appeared as if it was working overtime or moonlighting, as some would call it. However, working overtime contributed to these participants disrupting their normal sleeping patterns; thus, the three verbatim quotes present a narrative indicating that the participants felt that stopping working overtime would provide them more time for themselves and better-quality sleep.

The following participants shared their reality with self-care practices and what interventions might assist them in promoting self-care practices:

‘Uh, I feel guilty and irresponsible because I don’t get enough sleep in. Yes, work is busy, but I also work somewhere else after work, even on my annual leave days, Sundays, or, let me say, my entire off days. For me, I will prioritise cutting off moonlighting because it affects my sleep; in fact, I sleep very little, never a full 8-hour or 7 hours of sleep, and as a result, I am always tired and irritable.’ (Participant 10, 32 years old, male)

‘I work an 8-hour shift daily, which amounts to 40 hours every week, so I am always exhausted. So, after this discussion on self-care practices, I realised that I need to allocate time for myself on weekends to rest because as much as I am not working on weekends, I would moonlight at private hospitals to make extra cash for school fees for my brother’s children. I am also expected to assist my uncle and aunt with food every month even though they get monthly pensions from the government, and other family members also assist. I would have to live within my means.’ (Participant 7, 49 years old, female)

‘I work for an agency after work, which most of the time is night shift, so I would go home and sleep for an hour or less, then night shift starts, so I think I must stop working these extra hours so that I can sleep. I don’t know when last I had an 8 hours’ sleep, so I believe I need to start with a good sleeping pattern to feel good and energised.’ (Participant 4, 40 years old, male)

#### Sub-theme 3: Practise healthy eating habits to promote self-care practices

This sub-theme mirrored the participants’ desire to engage in self-care practices, including engaging in healthy eating habits, which would indirectly assist them with losing weight and preventing any complications from unhealthy eating habits.

The participants viewed healthy eating habits as imperative in contributing to self-care practices. Participants stated:

‘Yes, eating healthy will motivate me to eat all the right food and burn some calories; in return, I will feel good and be eager to practice self-care. I am a bit overweight because even after the divorce, I engaged in unhealthy eating habits, so these extra kilos must go.’ (Participant 6, 39 years old, male)

‘There is simply no time to make healthy meals, for a lunch box, eating unhealthily has become so easy because unhealthy is easy to find, and tastes very nice. But these kinds of food makes me to gain unwanted weight, which promotes a decrease in my self-esteem and causes me some bloating, so I will try to eat some green vegetables, cut on soda, and drink water; but I will give myself a body goal for weight loss, and if I struggled, I would need to consult a dietician, but this time I will try.’ (Participant 9, 30 years old, male)

‘I live alone, so unhealthy eating habits have been a practice I have been engaging in for the longest time in my life, which is the main reason I am so overweight, or at least what my body mass index indicates, but also my last cholesterol was not within normal limits. Hence, I would then say eating healthily, decreasing Coke and any bad soda will be what I suggest is vital or what I would choose to do to strive towards self-care practices that will make me lose some weight and decrease or eliminate possible complications related to unhealthy habits.’ (Participant 5, 51 years old, female)

#### Sub-theme 4: Promoting self-care practices by engaging in physical activity

Aside from the above-mentioned individual interventions regarding what can be done to promote self-care practices, the participants emphasised the importance of physical activity in contributing to self-care practices. The following verbatim quotes indicate what the participants viewed as important in practising self-care; they realised the importance of being physically active, including using a gym or walking. The participants shared the following:

‘I am glad I met the criteria to be part of this study because I realise, I have been neglecting myself, and the excuse has been I am busy, so I am going back to the gym, which is 2 minutes away from my house, and it closes at 21h00. Exercises, in general, make one to shred weight, releases all the good hormones, and also to have a good night’s sleep.’(Participant 4, 40 years old, male)

‘I have been promising myself to practise self-care but simply could not do it, so after today, no more excuses. But I am also realistic, so I will prioritise weight gain, which exercises are at the top of my list. Being physically active will assist me a lot.’ (Participant 1, 34 years old, female)

#### Sub-theme 5: To promote mental wellness through debriefing and employee wellbeing programmes

The findings of this study explicitly revealed the extent to which self-care neglect negatively impacted the participants’ mental wellbeing; however, they were eager to commit and participate in different interventions such as debriefing sessions, other continued mental wellbeing programmes and the available employee wellbeing programmes within their work environment. The following verbatim quotes give more insight into this sub-theme:

‘As nurses, we deal with so many patients daily, and it is not about examining them or giving them medication. We hear their daily life struggles, which causes some emotional discomfort in nurses’ lives. Therefore, I think I will use my medical aid or ask for a referral from my direct supervisor to talk to a psychologist or anyone that I can offload and debrief.’ (Participant 5, 51 years old, female)

‘During the peak of COVID-19, many of our colleagues died because of the COVID-19 pandemic, which promoted great anxiety, depression and great fear, and sadness in many nurses, so I think continued programmes within the workplace that deal with mental well-being and wellness is imperative to promote self-care practices.’ (Participant 10, 32 yeas old, male)

‘I divorced, had one miscarriage, and just recently lost a family member and two colleagues to COVID-19, the pandemic; these phases of my life have been difficult and painful, so what helped me to be mentally strong was making use of the employee well-being programme at work. It’s free of charge, so I will continue to use the employee well-being programme as a self-care practice, because taking care of your mental well-being is like taking care of your health because mental pressures can lead to severe depression, possibly leading to suicide.’ (Participant 8, 37 years old, female)

## Discussion

The results of this study revealed that professional nurses were mindful that they were not practising self-care and felt the urge to take responsibility for making self-care possible. In this study, the participants shared strategies they perceived as helpful for engaging in self-care with the researchers. These strategies contained interventions promoting their mental, physical and general well-being. This aligns with the study done by Butler et al. (2019), which states that self-care activities promote and maintain an individual’s physical, psychological and emotional well-being to achieve holistic well-being. Lee and Jovic (2018) affirm that despite challenges, such as long working hours, staff shortages and other limited resources within primary healthcare facilities, professional nurses should attempt to engage in self-care practices by nurturing their physical and psychological health.

In this study, the participants shared that they would attempt to engage in self-care by doing activities such as reading their favourite book, listening to their favourite music, or just engaging in activities they enjoyed. Schnitzer (2019) supports this view by stating that self-care activities that an individual enjoys (music, gardening, reading a book) will assist professional nurses in reducing possible stressors and anxiety they might experience in their work environment and personal lives. In addition, a study conducted within a hospital setting found that activities such as music, reading and adequate rest are essential to promote self-care among professional nurses and other health professionals (Lubinska-Welch et al. 2016). Toomey (2021) further unveils that these activities seem easy. Still, the majority of professional nurses in different clinical settings do not engage in such activities but would typically advise their patients or others to engage in such activities to promote their well-being. Similarly, a study done by Chipu (2021) revealed that professional nurses need to be mindful of how imperative these activities are to promote self-care practices and that professional nurses need to be disciplined and consistent in self-care practices.

In addition, the participants shared that they did not have an adequate sleep routine; thus, they saw it fitting to prioritise sleeping to ensure they rest; the inadequate sleeping pattern seemed to be attributed to the participants working overtime in healthcare settings other than their employed setting. Eanes (2015) highlighted that inadequate sleeping patterns occur when an individual sleeps for fewer than 7 h or 8 h, which negatively impacts memory and concentration levels, leaving the individual exhausted and frustrated. A quantitative study done by Muhlare (2022), which focused on self-care practices by professional nurses within primary healthcare settings, revealed that only 44% of professional nurses were adhering to a sleeping routine between 7 and 8 h, while 66% of professional nurses were not adhering to a 7–8 h sleeping routine. Chang and Jie (2023) stated that an adequate sleeping routine is essential to promote self-care practices among professional nurses. Self-care practices among professional nurses should include adequate sleeping patterns, as adequate sleeping patterns will not only promote health for professional nurses but will also promote patient safety and individual care (Di Simone et al. 2020). Professionals who encounter inadequate sleep are prone to commit medical errors (Johnson et al. 2014; Zhou et al. 2020). Udoudo et al. (2023) commented that, despite other activities that are imperative for self-care, adequate sleep is essential.

The participants indicated that they would work at other healthcare institutions on their days off or after work to earn supplementary income. Alibudbud (2024) revealed that professional nurses should minimise working overtime, despite the valid reasons they may have, as extended hours reduce the time available for them to engage in self-care practices. This statement was supported by Sehume (2016), who noted that overtime among professional nurses contributed to their suffering from ill health and should be prevented or minimised. Els (2017) advised that nurses should rest when they are not supposed to be on duty.

The participants emphasised that they would improve their dietary practices to enhance self-care. Davies (2020) confirmed this view by suggesting professional nurses prioritise good eating habits to improve their physical health. Professional nurses are prone to being overweight or obese because of poor dietary habits (Ross et al. 2019). The participants in this study alluded that their dietary practices contributed to weight gain and, ultimately, obesity; therefore, they wanted to start correcting their relationship with food. Torquati et al. (2018) asserted that poor nutritional intake among professional nurses not only hurt their weight but also made them susceptible to chronic conditions such as hypertension and diabetes. Power (2018) stated that to promote effective nutritional practices among professional nurses to promote self-care, professional nurses need continuous practical guidance on good dietary practices. Furthermore, a study done by Power et al. (2014) revealed that professional nurses need to enhance their dietary knowledge bases to make informed decisions about their dietary practices. Despite the importance of knowledge to promote effective dietary practices, Nicholls et al. (2016) argued that the work environment contributes to professional nurses not engaging in good nutritional habits, and this can only be corrected if professional nurses are supported in terms of the correct nurse-patient ratio, and the provision of all relevant resources.

Davies (2024) asserted that management at all levels is responsible for fostering a conducive environment to make self-practices attainable. This view is supported by the reality that a positive work environment with good nutritional values and work cultures will promote good nutritional values among professional nurses (Abujaber & Nashwan 2024). Healthcare institutions, such as hospitals and primary health clinics, should prioritise healthier food options, including fruits, vegetables and healthy sandwiches or wraps in cafeterias and vending machines, as current offerings often lean towards unhealthy choices (Horton Dias et al. 2022). Moreover, the participants viewed engaging in physical activities as a suitable form of self-care as they seemed not to prioritise physical activities that were beneficial to their health. Professional nurses should engage in intense or moderate exercise for at least 30 min to promote their physical appearance and well-being (Daud et al. 2021).

Dwivedi et al. (2015) divulged that intervention strategies such as yoga can be beneficial to individual professional nurses by improving their flexibility, balance and muscle tone. Professional nurses spend most of their time at work providing patient care. Hence, healthcare institutions should invest in promoting physical activities (gym facilities, physical activities) at work, ensuring they are easily accessible to all healthcare workers (Monakali et al. 2018). Torquati (2016) further concluded that physical activities should be promoted within healthcare facilities by nominating a champion to facilitate physical activities. Masfi et al. (2024) and Persby (2016) advised that professional nurses need to be emotionally ready to engage in physical activities such as jogging, walking, or brisk walking, as these activities require commitment.

Lastly, the participants stated that they were neglecting their mental well-being, and as a result, they had an inner desire to prioritise their mental well-being. Gibbons, Lauder and Ludwick (2017) outlined the importance of mental wellness and revealed that if professional nurses do not prioritise their mental well-being, it will affect various aspects of their lives. This statement is supported by a study that showed that because of self-care neglect, professional nurses suffered from depression, anxiety and severe stress (Melnyk et al. 2018). To uphold professional nurses’ mental well-being, they need to prioritise going for counselling as they are exposed daily to various factors that affect their mental well-being (Walton, Murray & Christian 2020). Greenberg, Wessely and Wykes (2015) concluded that professional nurses are aware of the importance of seeking mental health services, but because of perceived stigma, they do not make use of those services.

### Limitations of the study

Despite its importance, this study focused solely on professional nurses, excluding other categories of nursing staff, such as enrolled nurses and nursing auxiliaries. Critical information could have been omitted, as the lived experiences of the other nursing staff are excluded from this study.

### Recommendations

Although this study focused on recommendations for facilitating self-care practices, other stakeholders also need to contribute to making self-care practices a reality.

#### Nursing practice

To promote self-care activities as part of earning Continuing Professional Development (CPD) points. Daily, weekly and monthly in-service training on topics relating to self-care interventions, sufficient rest, physical exercise, the importance of eating healthily and the importance of mental wellness and mental well-being.

#### Nursing education

The described recommendations should be incorporated into the curriculum of professional nurses to facilitate their empowerment to practise self-care as a lifestyle, instil knowledge and create awareness of what needs to be done to practise self-care.

#### Nursing research

Further qualitative or quantitative research may be conducted that includes other categories of nursing personnel. In addition, the researchers may consider conducting a follow-up study to ascertain how the participants are implementing the stated interventions and their impact.

## Conclusion

The results of this study explicitly revealed the professional nurses’ desire to practise self-care. They shared with the researchers their intentions to make self-care practices a reality, as they could see the negative impact of self-care neglect: they constantly felt exhausted because of not resting, as they continually prioritised working overtime, engaging in unhealthy eating habits which led to undesired weight gain, and depriving themselves of activities such as reading, listening to music and even physical activities such as attending the gym. Furthermore, the results indicated that most perceived interventions to promote self-care practices are cost-effective and dependent on the individual.
